# A Combined Region- and Pixel-Based Deep Learning Approach for Quantifying Abdominal Adipose Tissue in Adolescents Using Dixon Magnetic Resonance Imaging

**DOI:** 10.3390/tomography9010012

**Published:** 2023-01-15

**Authors:** Olanrewaju A. Ogunleye, Harish Raviprakash, Ashlee M. Simmons, Rhasaan T.M. Bovell, Pedro E. Martinez, Jack A. Yanovski, Karen F. Berman, Peter J. Schmidt, Elizabeth C. Jones, Hadi Bagheri, Nadia M. Biassou, Li-Yueh Hsu

**Affiliations:** 1 Radiology and Imaging Sciences, Clinical Center, National Institutes of Health, Bethesda, MD 20892, USA; 2 Behavioral Endocrinology Branch, Division of Intramural Research, National Institute of Mental Health, National Institutes of Health, Bethesda, MD 20892, USA; 3 Section on Growth and Obesity, Division of Intramural Research, Eunice Kennedy Shriver National Institute of Child Health and Human Development, National Institutes of Health, Bethesda, MD 20892, USA; 4 Clinical and Translational Neuroscience Branch, Division of Intramural Research, National Institute of Mental Health, National Institutes of Health, Bethesda, MD 20892, USA

**Keywords:** abdominal adipose tissue, magnetic resonance imaging, fat quantification, segmentation, convolutional neural network

## Abstract

Background: The development of adipose tissue during adolescence may provide valuable insights into obesity-associated diseases. We propose an automated convolutional neural network (CNN) approach using Dixon-based magnetic resonance imaging (MRI) to quantity abdominal subcutaneous adipose tissue (SAT) and visceral adipose tissue (VAT) in children and adolescents. Methods: 474 abdominal Dixon MRI scans of 136 young healthy volunteers (aged 8–18) were included in this study. For each scan, an axial fat-only Dixon image located at the L2–L3 disc space and another image at the L4–L5 disc space were selected for quantification. For each image, an outer and an inner region around the abdomen wall, as well as SAT and VAT pixel masks, were generated by expert readers as reference standards. A standard U-Net CNN architecture was then used to train two models: one for region segmentation and one for fat pixel classification. The performance was evaluated using the dice similarity coefficient (DSC) with fivefold cross-validation, and by Pearson correlation and the Student’s t-test against the reference standards. Results: For the DSC results, means and standard deviations of the outer region, inner region, SAT, and VAT comparisons were 0.974 ± 0.026, 0.997 ± 0.003, 0.981 ± 0.025, and 0.932 ± 0.047, respectively. Pearson coefficients were 1.000 for both outer and inner regions, and 1.000 and 0.982 for SAT and VAT comparisons, respectively (all *p* = NS). Conclusion: These results show that our method not only provides excellent agreement with the reference SAT and VAT measurements, but also accurate abdominal wall region segmentation. The proposed combined region- and pixel-based CNN approach provides automated abdominal wall segmentation as well as SAT and VAT quantification with Dixon MRI and enables objective longitudinal assessment of adipose tissues in children during adolescence.

## 1. Introduction

Human adipose tissue has been described as an organ with both endocrine and metabolic functions. It acts as the body’s energy reserve and plays an important role in energy metabolism, neuroendocrine function, and immune function [1,2,3]. The overabundance of body fat is a significant global public health challenge that may lead to chronic disorders and impact quality of life [4]. Particularly, abdominal obesity is associated with medical conditions such as hypertension, dyslipidemia, and type 2 diabetes in people of all ages, including young adults and children [5]. Abdominal adipose tissue distribution and body composition may also predict cardiovascular risk and subsequent events [6].

Childhood obesity is a global phenomenon affecting all socio-economic groups, irrespective of age, sex, or ethnicity [7]. Childhood and adolescent obesity have increased in the past five decades and are associated with an increased risk of obesity in adults. Many children and adolescents with obesity already manifest some metabolic complications, and these children are at high risk for the development of early morbidity [8]. Children and young people who have obesity are more likely to develop obesity-related cardiovascular and metabolic diseases in their lifetime [4,9]. Therefore, fat quantification in early life may be used for initial assessments of significant risks of cardiovascular disease [10,11].

Abdominal adipose tissue is distributed in different depots, including visceral tissue, subcutaneous tissue, muscles, and organs such as breast and bone marrow. Visceral adipose tissue (VAT) and subcutaneous adipose tissue (SAT) are most frequently studied due to their association with health risks. Furthermore, VAT and SAT have different risk profiles, making it important for clinicians to stratify patients according to their obesity phenotypes, i.e., VAT versus SAT [12].

Existing studies show that the increased accumulation of VAT is more strongly related to a poor metabolic and inflammatory profile in humans than SAT [5,13]. SAT shows a greater activity for long-term energy storage, whereas VAT exhibits greater metabolic and hormonal activity through the release of adipokines [14,15]. This may be related to the fact that visceral adipocytes have greater lipolytic activity than subcutaneous adipocytes, and VAT has a larger impact on the free fatty acid level in the systemic circulation than other abdominal fat depots [14,16].

Different techniques have been utilized for body fat measurement, each with its advantages and shortcomings. Anthropometry, such as waist circumference and body mass index, has the benefit of being non-invasive and easy to learn and utilize for estimating body fat [17]. However, it relies on derived equations that are dependent on variable factors such as age, gender, and ethnicity, making it potentially inaccurate [18]. Two-dimensional (2D) projections of the body using dual-energy X-ray absorptiometry (DEXA) is accurate for estimating whole-body fat; however, a major drawback is that it has limited performance for determining compartmental fat volume when compared with 2D cross-sectional or three-dimensional (3D) volumetric techniques [19].

Computed tomography (CT) can generate accurate cross-sectional images for the localization of compartmental fat, but a major disadvantage is its utilization of potentially hazardous ionizing radiation, limiting its use for this purpose. There are several advantages to the use of magnetic resonance imaging (MRI) as a fat measurement method over other fat measurement techniques including anthropometry, DEXA, and CT. These include the better characterization of different tissue types, non-invasive nature, and the absence of ionizing radiation. In MRI, the detailed localization of compartmental fat is achieved because of high inherent contrasts on soft tissue imaging.

On a T1-weighted MRI, there is good differentiation of signal intensity contrast between adipose tissue, which appears bright, and other soft tissues such as muscles, which appear darker. The dual-echo T1 imaging using the Dixon-based approach provides greater differentiation of adipose versus other soft tissues due to its fat-and-water separation technique [20,21]. The Dixon method uses a modified spin echo pulse sequence to acquire in-phase and out-of-phase images. A fat-only Dixon image can then be generated by the difference between the two images.

In this study, we aimed to develop a machine-learning-based convolutional neural network (CNN) approach that combined both region- and pixel-based segmentation for the automated quantification of subcutaneous adipose tissue (SAT) and visceral adipose tissue (VAT) on Dixon-based MRI, with the goal of achieving high accuracy for longitudinal assessments of adipose tissues in children and adolescents.

## 2. Materials and Methods

### 2.1. Study Participants

Abdominal MR imaging was obtained from young healthy volunteers whose pubertal status was assessed, and in whom endocrine and metabolic measures were evaluated at eight- to ten-month intervals from an age of 8 years (pre-puberty) until an age of 18 years (post-puberty). All participants were enrolled under an ongoing protocol (11M0251/NCT01434368) to study reproductive endocrine, metabolic, and physical measures and to characterize the stage and duration of pubertal development [22].

All MR studies were performed under procedures and protocols approved by the Institutional Review Board of the National Institutes of Health. Written informed assent was obtained from all children in combination with parents’/legal guardians’ written consent prior to participating in the study.

### 2.2. MRI Image Acquisition

The MRI image acquisition was performed at the Radiology and Imaging Sciences department of the National Institutes of Health Clinical Center. All abdominal scans in this study were acquired on a 3T whole-body MRI scanner (Philips Medical Systems, Best, The Netherlands) using a SENSE XL Torso receiving coil for signal reception. A standard three-dimensional two-point Dixon T1-weighted imaging sequence was prescribed with typical acquisition parameters of repetition time 3.41 ms, echo times 1.19 ms and 2.37 ms, flip angle 10°, pixel bandwidth 1965 Hz/pixel, percent phase field of view 72.2, field of view 317 mm × 317 mm, acquisition matrix 212 × 212, and reconstruction image matrix 288 × 288. Two image series were acquired to cover the L2–L3 and L4–L5 spine segments separately.

### 2.3. Image Analysis

For each image series, an axial slice location was selected based on the sagittal localizer image at the center of L2–L3 and L4–L5 levels. This analysis protocol was established for the longitudinal assessment of the progression of abdominal adipose tissues in children during adolescence. Next, the axial fat-only Dixon image was used to generate initial labels based on semi-automated medical image analysis software developed in-house to contour abdominal walls and threshold bright fat pixels based on 50% of the maximum signal intensity, followed by manual correction. For each image, outer and inner regions around the abdomen wall, as well as SAT and VAT pixel masks, were labeled. Overall, there were 765 images labeled by a radiologist with 3 years of experience (O.A.O.) and a research assistant with 2 years of experience in image processing (A.M.S.). The final results were then reviewed by another radiologist with 28 years of experience (HB).

**Figure 1:**
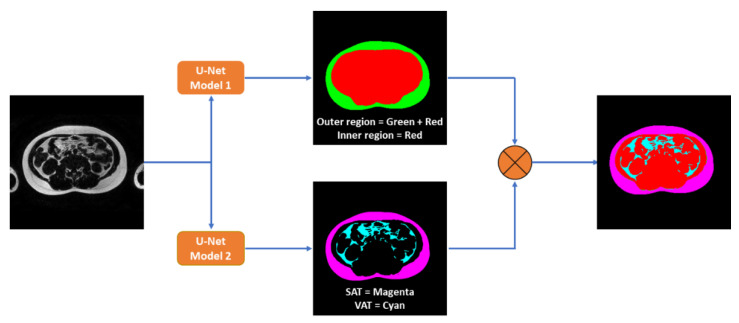
A standard U-Net architecture was used to train two models: one for outer and inner abdominal wall region segmentation, and one for subcutaneous (SAT) and visceral (VAT) adipose tissue pixel classification.

The software allows the user to edit the outer and inner contours as well as remove false-positive pixels through an interactive graphical user interface, such as excluding islands of fat within and around the spinal canal and the neural foraminal. The software also allows region-to-contour and contour-to-region conversions. These four types of masks served as the reference labels for CNN training and testing, as shown in Figure 1.

### 2.4. CNN Approach

A standard U-Net architecture with 4 layers in the encoder and the decoder was implemented for the segmentation tasks [23]. The overview of our proposed approach is shown in Figure 1. Two independent models were trained using the generalized Wasserstein distance loss [24]: one for region segmentation and one for fat pixel classification. The outputs of the two models were then combined to generate the 4 class labels: outer region, inner region, SAT, and VAT. The final performance was evaluated using the dice similarity coefficient (DSC).

The algorithm was implemented in PyTorch using an NVIDIA Quadro RTX 5000 GPU card. Input images of size 384 x 384, with pixel values normalized to a range between 0 and 1, were used to train the network with a batch size of 8. An Adam optimizer with an initial learning rate of 0.0001 and a learning rate scheduler with a patience of 10 epochs was used to train the model for 100 epochs. Fivefold cross-validation was performed to estimate the robustness of the proposed algorithm. The results of the testing from each of the five folds were averaged for reporting.

### 2.5. Statistical Analysis

Outer and inner abdomen region segmentation, as well as VAT and SAT pixel classification were evaluated separately. Agreement between the reference and computer-generated results was evaluated using the Pearson correlation coefficient and Bland–Altman plot. A Student’s t-test was used to determine statistical significance. A *p*-value < 0.05 was considered statistically significant for each test. The results are shown as the mean ± standard deviation (SD).

## 3. Results

A sample of 136 participants, 78 boys and 58 girls, 474 abdominal Dixon MRI scans in total, were included in this study. As shown in Table 1, the mean age was 11 years (range 8–18), the mean BMI was 18.19 kg/m^2^ (ranges from 13.20 to 25.19 kg/m^2^); other demographic information of the participants is summarized in Table 1.

**Figure 2:**
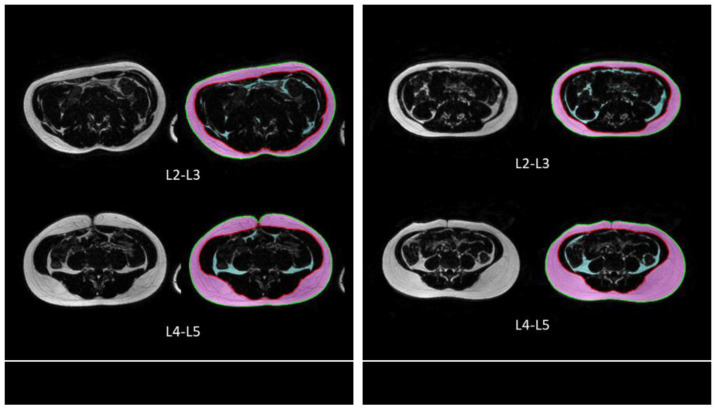
Two example cases showing the qualitative results of the proposed technique detecting the outer abdominal region (converted to green contour) and inner abdominal region (converted to red contour), as well as subcutaneous adipose tissue (magenta pixels) and visceral adipose tissue (cyan pixels).

For qualitative comparison, Figure 2 shows two example cases of the proposed technique for segmenting the outer and inner abdominal regions, as well as quantifying SAT and VAT pixels, from the axial images of the abdomen at L2–L3 and L4–L5 locations. For quantitative performance evaluation of the overall results, the means and SDs of DSC for segmenting the outer region and inner region were 0.974 ± 0.026 and 0.997 ± 0.003, respectively. For SAT and VAT quantification, the means and SDs of DSC were 0.981 ± 0.025 and 0.932 ± 0.047, respectively.

Separate comparisons of the segmentation results at different disc locations were also performed. At the L2–L3 location, the means and SDs of DSC were 0.968 ± 0.029, 0.997 ± 0.002, 0.977 ± 0.030, and 0.920 ± 0.047 for the outer region, inner region, SAT, and VAT, respectively. At the L4–L5 location, the means and SDs of DSC were 0.981 ± 0.020, 0.997 ± 0.004, 0.986 ± 0.018, and 0.946 ± 0.044 for the outer region, inner region, SAT, and VAT, respectively. The performance was greater for segmenting the axial image at the level of L4–L5 than the level of L2–L3 for the outer region, as well as SAT and VAT (all *p* < 0.01). However, the performance was not statistically significantly (NS) different for segmenting the inner region between the two locations of L2–L3 and L4–L5 (p = NS).

**Figure 3:**
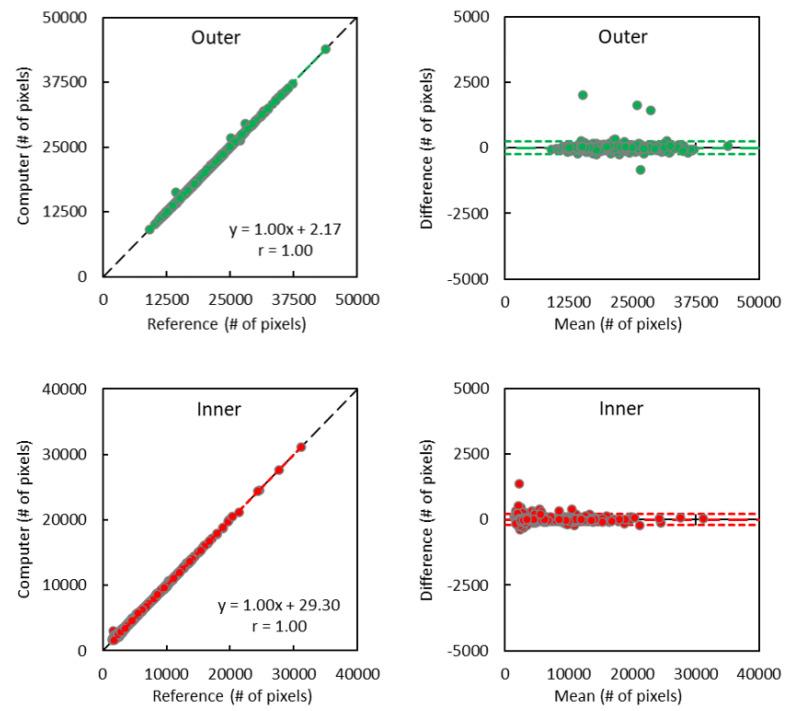
Pearson coefficients and Bland–Altman plots show excellent correlations and agreements between computer (convolutional neural network approach) versus reference (human) segmentation of the outer (green) and inner (red) abdomen regions. The dashed and dotted lines in Bland–Alman plots represent the bias and limits of agreement, respectively (mean ± 1.96 SD).

**Figure 4:**
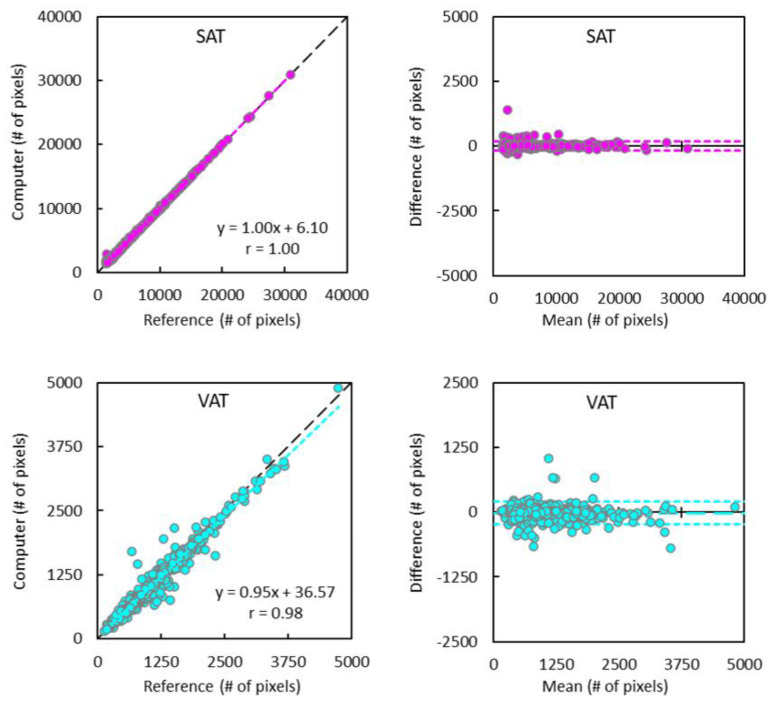
Pearson coefficients and Bland–Altman plots show excellent correlations and good agreement between computer (convolutional neural network approach) and reference (human) segmentation of subcutaneous adipose tissue (SAT) and visceral adipose tissue (VAT). The dashed and dotted lines in Bland–Alman plots represent the bias and limits of agreement (mean ± 1.96 SD).

The linear regression and Bland–Altman analyses results of the computer CNN segmentation versus human reference labels are shown in Figure 3 and Figure 4, respectively. Overall, Pearson coefficients were 1.000 for both outer and inner regions, and 1.000 and 0.982 for SAT and VAT comparisons, respectively (all p = NS). Bland–Altman analyses revealed minimum biases with good limits of agreement in all comparisons.

## 4. Discussion

This is the first study to demonstrate that Dixon-based MRI provides excellent water–fat image contrast to depict abdominal adipose tissues in children and adolescents. We also proposed a fully automated four-label hybrid CNN-based method for the accurate quantification of abdominal adipose tissues in these young healthy study participants. Our results showed that this new combined pixel-based and region-based deep learning approach not only provides excellent agreement with the human-expert-generated reference SAT and VAT measurements; it also produces accurate abdominal wall region segmentation, allowing the user to modify the automated results using an alternative threshold-based approach if needed. Furthermore, the additional segmentation of abdominal wall regions provides a ratio of fat distribution or percentage to the abdominal trunk area or waist circumference. The proposed approach enables a robust and objective assessment of the changes in abdominal VAT and SAT during adolescence.

There are different approaches to quantifying body fat components from acquired MR images. A completely manual analysis is extremely time-consuming and not feasible in large studies, leading to a demand for automated methods [25]. Additionally, the automated analysis of body fat from the MR images has potentially higher precision because of reduced or eliminated dependency on human operator variability [26].

The widespread implementation of automatic and semi-automatic techniques based on intensity and shape features, such as fuzzy clustering, k-means clustering, graph-cuts, active contour methods, and statistical shape models has been limited due to difficulties in achieving good accuracy in complex abdominal adipose tissue structures. This difficulty arises from several factors; a wide variety of VAT shapes, large anatomical differences across subjects and the inherent properties of the Dixon images, such as low-intensity contrast between different classes of adipose tissue, inhomogeneous signals, and potential organ motion [27,28,29,30,31,32].

Convolutional neural networks (CNNs) have become the state of the art for image segmentation. They are used for pixel/voxel-wise image segmentation in an end-to-end fashion to overcome challenges encountered by manual segmentation. CNNs can automatically extract intrinsic features and integrate global context to resolve local ambiguities, thereby improving the results of the predicted models [33]. CNN models have shown successful applications for SAT and VAT quantification on whole-body MRI volumes [10,33,34]. Our CNN was designed to generate both region- and pixel-based labels for SAT and VAT quantification. Not only does it produce pixel-based labels for SAT and VAT, but it also generates inner- and outer-region-based labels of the area to study which allows the user to select an alternative threshold-based approach to overwrite the SAT and VAT labels in case the CNN results are suboptimal.

Our result is comparable to similar findings by other authors that utilized the U-Net architecture to measure adipose tissue on MRI images. Kway et al. reported that the automated 2D segmentation U-Net architecture can correctly exclude non-fat structures in the abdomen and achieve accurate volumetric fat assessments [10]. Langer et al. reported that a U-Net based strategy of fat quantification can generate accurate and robust automated VAT and SAT segmentations [34]. They further compared two network architectures and concluded that U-Net is more robust and outperformed the V-Net architecture [34]. Our U-Net also produced a better Dice score when compared with the FatSegNet architecture developed by Estrada et al. [33] (0.932 vs. 0.850 for VAT, and 0.981 vs. 0.975 for SAT), despite using a different dataset.

A similar study quantifying abdominal adipose tissues in children and adolescents was presented recently [11]. In that study, a single MRI slice at the level of the second lumbar vertebra (L2) was selected for a longitudinal assessment of adipose tissue in healthy subjects aged from 6 to 18 years. However, abdominal SAT and VAT compositions in the study were quantified from T2-weighted MRI images which had a less fat-specific tissue contrast than the T1-weighted Dixon MRI used in our study. Furthermore, their study used semi-automated quantification software with an average analysis time between 5 and 15 min for each image.

In this study, we studied young healthy volunteers between the ages of 8 and 18 years old, because research shows that intra-abdominal adipose fat accumulation begins in this unique age group and is associated with significant adverse health effects occurring in obese children and adolescents either before or after they become adults [35,36]. Similar to the prior study by Marunowski et al. [11], our fat quantification was focused on cross-sectional images at the level of L2–L3 and L4–L5 interspaces due to the considerable changes in physical and body composition during adolescence. Nevertheless, previous studies have shown that the adipose tissue area quantified from a single-slice image sampled at various intervertebral disc levels had strong correlations with total abdominal and visceral adipose tissue volumes [37,38].

The present study has some limitations. First, our dataset in the current study only included pediatric participants who underwent repeated MRI scans during adolescence at a single institution. A new training dataset may be required before applying our method to quantify abdominal fat from adult MR images. Second, our analysis was based on quantifying cross-sectional 2D slices to follow up the growth and development of adipose tissues in young healthy volunteers. Additional images and labels at different slice locations will be required to train the CNN before applying it to full tomographic MR image stacks. Finally, image artifacts are an important issue in MR imaging. Although the Dixon technique has been improved extensively in the aspects of phase errors, noise, and artifacts [39], image artifacts may inevitably affect the accuracy of our fat quantification method.

## 5. Conclusions

Dixon-based MRI is an effective technique for measuring adipose tissue in children and adolescents due to its excellent fat tissue contrast. The proposed automated CNN approach provides comprehensive abdominal wall segmentation as well as SAT and VAT quantification for an objective longitudinal assessment of adipose tissues in children during adolescence.

## Figures and Tables

**Table 1:** Demographic information for the study participants undergoing abdominal MRI for fat quantification (SD: standard deviation, SEM: standard error of the mean, BMI: body mass index).

**Participants = 136**						
Gender	Boy	Girl				
	78	58				
Race/Ethnicity	White	Black	Asian	Hispanic	Mixed	Other
	68	23	12	10	14	9
**MRI Scans = 474**						
Range	Mean	SD	SEM	Min	Max	
Age (years)	11	3	0.12	8	18	
Height (cm)	149.98	15.43	0.71	121.10	190.00	
Weight (kg)	42.12	13.38	0.61	20.10	89.50	
BMI (kg/m^2^)	18.19	2.34	0.11	13.20	25.19	
BMI SD Score *	0.14	0.62	0.03	−1.98	1.58	
BMI Percentile * (%)	54.77	21.48	0.99	2.37	94.33	
Body Fat Percentage † (%)	26.01	5.87	0.28	13.67	46.61	

* Based on the Centers for Disease Control and Prevention 2000 growth charts. † Measured by dual-energy X-ray absorptiometry (Hologic, Inc, Bedford, MA).

## Data Availability

The dataset presented in this article is not readily available due to patient confidentiality. Requests to access the dataset should be directed to the corresponding author.

## Abbreviations

2D	two-dimensional
3D	three-dimensional
BMI	body mass index
CNN	convolutional neural network
CT	computed tomography
DEXA	dual-energy X-ray absorptiometry
DSC	dice similarity coefficient
MRI	magnetic resonance imaging
NS	not statistically significant
SAT	subcutaneous adipose tissue
SD	standard deviation
SEM	standard error of the mean
VAT	visceral adipose tissue
